# Nano‐calcipotriol as a potent anti‐hepatic fibrosis agent

**DOI:** 10.1002/mco2.354

**Published:** 2023-08-26

**Authors:** Yina Zhang, Liying Wang, Jiajia Shao, Yanning Liu, Yining Lu, Jing Yang, Siduo Xu, Jingkang Zhang, Minwei Li, Xiangrui Liu, Min Zheng

**Affiliations:** ^1^ State Key Laboratory for Diagnosis and Treatment of Infectious Diseases National Clinical Research Center for Infectious Diseases Collaborative Innovation Center for Diagnosis and Treatment of Infectious Diseases The First Affiliated Hospital College of Medicine Zhejiang University Hangzhou China; ^2^ Department of Pharmacology and Department of Gastroenterology of the Second Affiliated Hospital Zhejiang University School of Medicine Hangzhou China; ^3^ Department of General Surgery Sir Run Run Shaw Hospital Zhejiang University School of Medicine Hangzhou China; ^4^ Key Laboratory of Biomass Chemical Engineering of Ministry of Education and Center for Bionanoengineering College of Chemical and Biological Engineering Zhejiang University Hangzhou China; ^5^ Cancer Center Zhejiang University Hangzhou China

**Keywords:** calcipotriol, hypercalcemia, liver fibrosis therapy, polymer micelle

## Abstract

Calcipotriol (CAL) has been widely studied as a fibrosis inhibitor and used to treat plaque psoriasis via transdermal administration. The clinical application of CAL to treat liver fibrosis is bottlenecked by its unsatisfactory pharmacokinetics, biodistribution, and side effects, such as hypercalcemia in patients. The exploration of CAL as a safe and effective antifibrotic agent remains a major challenge. Therefore, we rationally designed and synthesized a self‐assembled drug nanoparticle encapsulating CAL in its internal hydrophobic core for systematic injection (termed NPs/CAL) and further investigated the beneficial effect of the nanomaterial on liver fibrosis. C57BL/6 mice were used as the animal model, and human hepatic stellate cell line LX‐2 was used as the cellular model of hepatic fibrogenesis. Immunofluorescence staining, flow cytometry, western blotting, immunohistochemical staining, and in vitro imaging were used for evaluating the efficacy of NPs/CAL treatment. We found NPs/CAL can be quickly internalized in vitro, thus potently deactivating LX‐2 cells. In addition, NPs/CAL improved blood circulation and the accumulation of CAL in liver tissue. Importantly, NPs/CAL strongly contributed to the remission of liver fibrosis without inducing hypercalcemia. Overall, our work identifies a promising paradigm for the development of nanomaterial‐based agents for liver fibrosis therapy.

## Graphical abstract

In the present study, we rationally synthesized a self‐assembled drug nanoparticle encapsulating calcipotriol (CAL) in its internal hydrophobic core for systematic injection (termed NPs/CAL) and further characterized the synthesized polymers. We found significantly reduced expression of extracellular matrix and hepatic stellate cell (HSC) activation marker with the intravenous administration of NPs/CAL in a mouse model of liver fibrosis induced by carbon tetrachloride (CCl_4_).

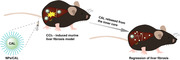

## 1 INTRODUCTION

Liver fibrosis represents a growing global health concern that is secondary to liver damage caused by multiple pathogenic factors. Chronic infection with hepatitis B and C virus (HBV and HCV) is widely believed to be the main cause of hepatic fibrogenesis,^1^ in addition, alcoholics and patients who suffer from fatty liver and some rare diseases are also prone to liver fibrosis.^2^, ^3^, ^4^, ^5^ With progressive injury, the majority of patients with liver fibrosis will eventually develop cirrhosis, which leads to over one million deaths globally each year.^3^, ^6^ The most prominent hallmark of liver fibrosis is the overproduction of extracellular matrix (ECM) by activated hepatic stellate cells (HSCs).^7^ When triggered by various profibrotic factors, quiescent HSCs are transformed into myofibroblast‐like cells, which secrete excessive amounts of ECM and proinflammatory cytokines, resulting in hepatic fibrogenesis.^8^, ^9^ Currently, liver transplantation is the optimal method for end‐stage cirrhosis; however, the effectiveness and adaptability of liver transplantation are greatly limited.^10^ Moreover, a standard and coherent treatment regimen for liver fibrosis has not yet been approved.^11^

Calcipotriol (CAL), the most widely studied synthetic vitamin D analog, has nearly the same affinity for vitamin D receptor (VDR) as 1,25(OH)_2_D3.^12^ Recently, the antifibrotic activity of CAL has attracted much attention. A plethora of studies have shown that CAL can negatively regulate phenotypes associated with HSC activation to alleviate fibrotic lesions of the liver.^13^, ^14^, ^15^ Although CAL has a potential therapeutic effect, its systemic administration is usually less effective due to its short half‐life. The property of low water solubility causes CAL to lack an appropriate administration route. The widespread distribution of VDR also reduces the amount of CAL arriving at the targeted site and induces possible adverse effects, such as hypercalcemia. To date, few examples of nano‐based formulations of CAL for hepatic fibrosis therapy have been reported. Motivated by the application prospects in the clinical field along with the hypercalcemia of CAL in patients, we envisioned that a rational nanoengineering strategy could enable CAL to exert sufficient antifibrotic activity while reducing the systemic effects of the upregulation of blood calcium to a tolerable degree.

Therefore, we sought to synthesize a self‐assembled polymer nanocarrier delivering CAL (termed NPs/CAL). The key design feature of this polymer was that we found a planar and hydrophobic stabilizer, that was, cholesterol, and introduced it into the polymer framework to ensure efficient packaging of CAL. In this study, enabling insoluble CAL to become an injectable drug with the help of nanocarriers prolonged the blood circulation of CAL. At the tissular and cellular level, in addition to the high accumulation of NPs in the liver uncovered by near‐infrared fluorescence imaging, flow cytometry and confocal laser‐scanning microscopy (CLSM) also showed efficient uptake of NPs by the human HSC line LX‐2. Then, we found NPs/CAL had significant antifibrotic activity, both in vitro and a murine liver fibrosis model. Most notably, NPs/CAL, unlike the positive control CAL, did not cause hypercalcemia in the body. Therefore, the nano‐enabled approach that we designed and developed to encapsulate and deliver CAL may represent a promising paradigm as an effective liver fibrosis therapy, as evidenced by their high efficacy and safety.

## 2 RESULTS

### 2.1 Synthesis and characterization of NPs/CAL

**FIGURE 1:**
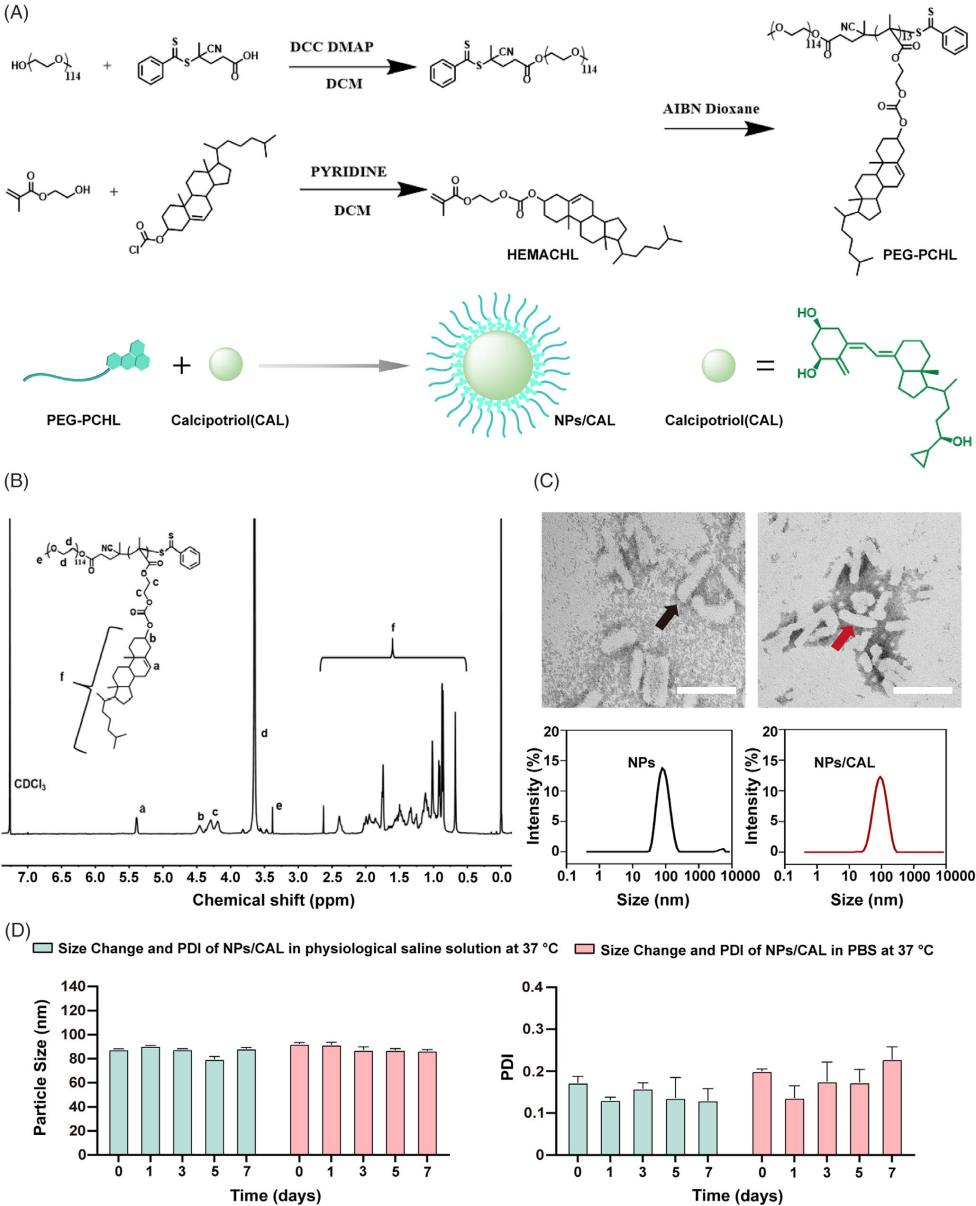
Synthesis and characterization of NPs/CAL. (A) Schematic overview of the synthesis of PEG_5K_‐P(HEMACHL)_7K_ and NPs/CAL. (B) The ^1^H‐NMR spectrum of PEG_5K_‐P(HEMACHL)_7K_. (CDCl_3_, δ ppm): 0.67–2.42 (m, ‐CH_3_, ‐CH(CH_3_)‐, ‐CH‐, ‐CH_2_‐), 3.37 (s, 3H, CH_3_‐O‐), 3.65 (s, 455H, ‐O‐CH_2_‐CH_2_‐O‐, ‐O‐CH_2_‐CH_2_‐OCO‐), 4.30 (m, 52H, ‐CO‐O‐CH_2_‐CH_2_‐O‐CO‐), 4.40–4.50 (m, 13H, ‐CHO‐), 5.37–5.40 (m, 13H, C = CH‐). (C) TEM images and size distributions of NPs and NPs/CAL. The black arrow on the left image and the red arrow on the right image refer to NPs and NPs/CAL, respectively. Scale bar = 100 nm. (D) The stability of the NPs/CAL in physiological saline solution and PBS at 37°C. TEM, transmission electron microscopy.

The reversible addition‐fragmentation chain transfer (RAFT) agent, 4‐Cyano‐4‐(2‐phenylethanesulfanyl‐thiocarbonyl) sulfanylpentanoic acid (PETTC), the macro‐RAFT agent, polyethylene glycol (PEG)‐PETTC, and the methacrylate ester of cholesterol (HEMACHL) monomer were synthesized first according to previous studies,^16^, ^17^ and the ^1^H‐NMR spectrum of cholesterol monomer is shown in Figure S1. The PEG_5K_‐P(HEMACHL)_7K_ amphiphilic polymer was synthesized by RAFT from the macro‐RAFT agent PEG‐PETTC and HEMACHL, and the detailed synthetic route of the amphiphilic polymer is shown in Figure 1A. The chemical composition of PEG_5K_‐P(HEMACHL)_7K_ was fully determined by ^1^H‐ NMR spectrum (Figure 1B) and gel permeation chromatography (GPC, Figure S2). The results enabled us to confirm the number of cholesterol monomers in each polymer, that was, 13 units of cholesterol domain in PEG_5K_‐P(HEMACHL)_7K_. According to a previous study by our laboratory,^16^ CAL could be loaded in the nanoparticle with the help of the cholesterol domain, and the solubility of CAL increased to 0.49 mg/mL. The NPs/CAL was fabricated with PEG_5K_‐P(HEMACHL)_7K_. The drug loading (DL) and encapsulation rate of NPs/CAL were approximately 5% and 87%, respectively. The critical micelle concentration (CMC) of the copolymer was 39.7688 μg/mL by Nile red method (Figure S3). Transmission electron microscopy (TEM) imaging revealed that NPs and NPs/CAL were uniformly rod‐like particles with an average diameter of 90 nm. The size and shape of the nanoparticles were not influenced after loading CAL (Figure 1C). The zeta potential of NPs was −16.93 mv and that of NPs/CAL was −9.89 mv (Figure S4), indicating that the loading of CAL was successful. Next, we evaluated the stability of NPs/CAL by mixing the nanoparticles with equal amounts of normal saline (sodium chloride solution, 0.9%, w/v) or phosphate‐buffered saline (PBS) (pH = 7.4) at 37°C. As shown in Figure 1D, the size and polydispersity (PDI) of NPs/CAL determined by dynamic light scattering (DLS) were basically unchanged over 7 days, indicating the good dimensional stability of the NPs/CAL in physiological condition. Then, we studied the release of CAL from the inner core in the Tris buffer at 37°C. in vitro drug release profile of NPs/CAL was performed by high‐performance liquid chromatography (HPLC) and the result showed that ∼30% CAL released from the nanoparticle at 24 h (Figure S5). In conclusion, we rationally designed and synthesized a self‐assembled drug nanoparticle encapsulating CAL in its internal hydrophobic core for systematic injection and further characterized the synthesized polymers by measuring the shape, size, stability, and drug release profile of Cal in the micelles.

### 2.2 NPs/CAL had good biocompatibility, high cell uptake efficiency, and significant in vitro antifibrosis effect

**FIGURE 2:**
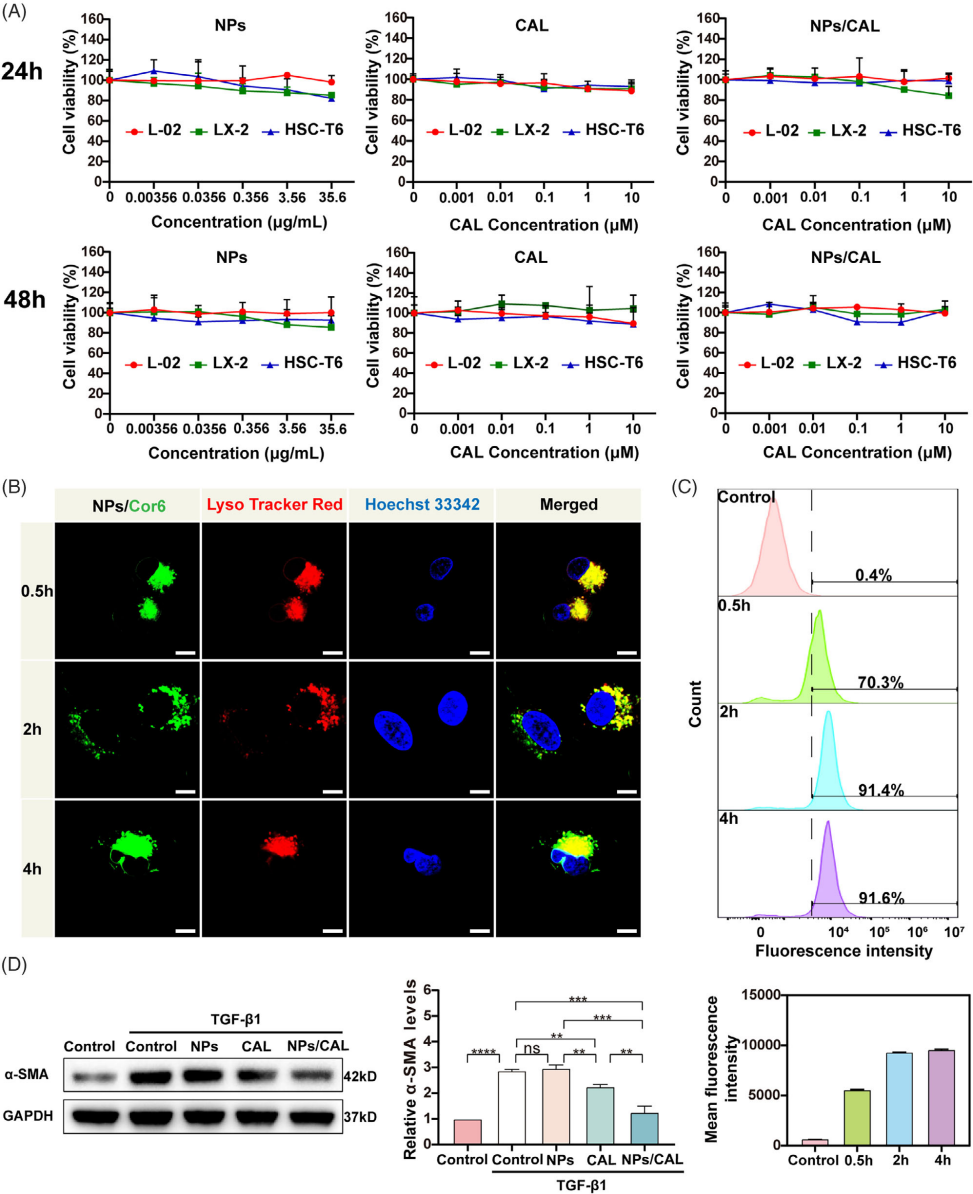
Assessment of cell viability, intracellular distribution, and cellular uptake of NPs and anti‐hepatic fibrosis activity of NPs/CAL in vitro. (A) Cytotoxicity of NPs, CAL, and NPs/CAL in L‐02, LX‐2, and HSC‐T6 cells at 24 and 48 h, respectively. All error bars correspond to mean ± SD (*n* = 3). (B) CLSM images of LX‐2 cells incubated with NPs/Cor6 for 0.5, 2, and 4 h. Cor6, lysosomes, and nucleus were shown in green, red, and blue, respectively. Scale bar = 10 μm. (C) Examination of the cell uptake efficiency of NPs/Cor6 in LX‐2 cells by flow cytometry. (D) Immunoblotting validation of α‐SMA protein in LX‐2 cells treated with CAL or NPs/CAL and TGF‐β1 for 48 h. CLSM, confocal laser‐scanning microscopy.

The cell viability assays of NPs, CAL, and NPs/CAL in normal hepatocyte line L‐02, LX‐2, and rat HSC line HSC‐T6 were determined by 3‐(4,5‐Dimethyl‐2‐thiazolyl)−2,5‐diphenyltetrazolium bromide (MTT) method (Figure 2A). When the concentration of NPs was 35.6 μg/mL, the viability of all three kinds of cells was still higher than 80% following a 24 or 48 h in vitro incubation, demonstrating the good biocompatibility of NPs at a high polymer concentration. The viability of all cell types treated with free CAL and NPs/CAL was further studied. It seemed that free CAL and NPs/CAL showed little cytotoxicity to all three cells which were treated with CAL at a concentration below 10 μM, indicating the good biocompatibility of NPs/CAL at these concentrations.

Next, choosing coumarin‐6 (Cor6) as a fluorescent dye, we then tested the intracellular distribution of the PEG_5K_‐P(HEMACHL)_7K_ amphiphilic polymer by using CLSM (Figure 2B). LysoTracker Red and Hoechst 33342 were able to label lysosomes/late endosomes and nucleus with red and blue pseudocolors, respectively. CLSM images clearly showed strong colocalization of Cor6 (green) with LysoTracker Red (red) after half an hour of incubation, which indicated that NPs/Cor6 entered the lysosome via the endocytic mechanism at a quick rate. After 2 h, we observed a faint green fluorescent signal in the cytoplasm of LX‐2 cells, indicating a subsequent fast release from the lysosome of Cor6 following internalization. Surprisingly, a stronger green fluorescent signal was observed throughout the cytoplasm another 2 h later. Hence, one can see that the NPs designed in this study not only had a high endocytosis speed but also exhibited a manifest intracellular release efficiency, which was particularly critical for NPs to transport CAL to achieve antifibrotic effects. We next attempted to comprehensively evaluate the efficiency of NPs/Cor6 uptake by LX‐2 using flow cytometric examination and found that the fluorescence intensity was detected in more than 90% of LX‐2 cells after a 2 h incubation (Figure 2C). in vitro, to define the inhibitory effects of NPs/CAL on fibrogenic protein induction by TGF‐β1 stimulation in HSCs, we conducted immunoblotting to verify the level of α‐smooth muscle actin (α‐SMA), a typical marker in activated HSCs (Figure 2D). As expected, incubation with CAL or NPs/CAL reduced TGF‐β1‐induced overexpression of α‐SMA, which revealed that CAL was able to restore activated HSCs to a resting state, whereas downregulation in α‐SMA expression was more significant after incubation with NPs/CAL, suggesting that NPs/CAL had good anti‐hepatic fibrosis efficacy in vitro. In summary, NPs/CAL exhibited good biological compatibility in vitro, and was able to be rapidly internalized by cells to display an excellent anti‐hepatic fibrosis effect.

### 2.3 NPs increased the accumulation of CAL in the liver and prolonged CAL circulation in the blood

Before studying the tissue distribution of NPs, we assessed the blood compatibility of NPs by a hemolytic activity assay (Figure S6). Compared with the positive control which resulted in complete lysis and no remaining red blood cells at the bottom of the tube, NPs caused no hemolysis and negligible erythrocyte membrane disturbance even if the concentration was as high as 100 μg/mL, indicating that NPs had high blood compatibility.

**FIGURE 3:**
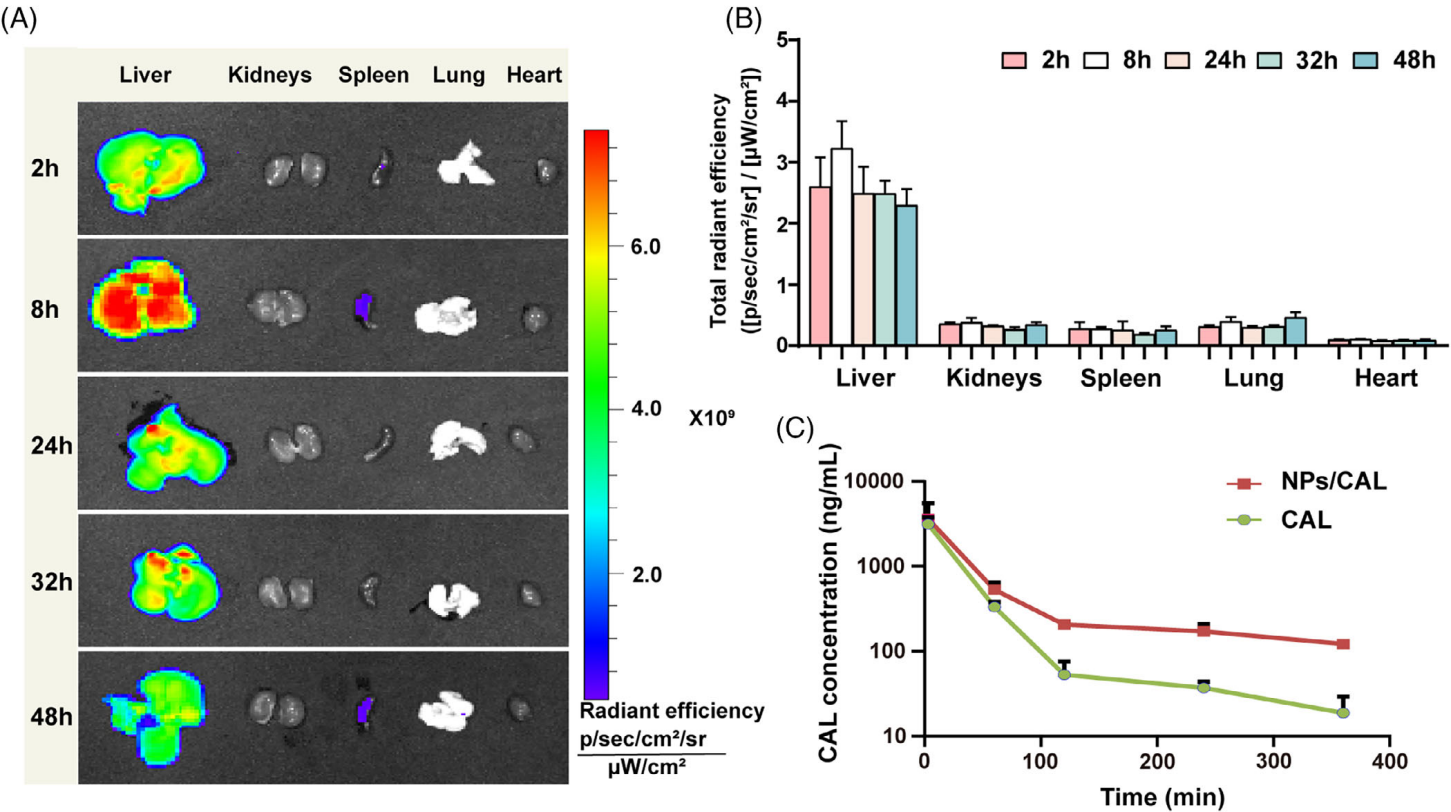
Biodistribution and blood clearance of NPs/CAL. (A) *Ex vivo* near‐infrared fluorescent images of five major organs harvested from normal mice at different time points post‐injection of NPs/DiI. (B) Representation of the fluorescence intensity of organs in the form of total radiant efficiency. All error bars correspond to the mean ± SD; *n* = 3/group. (C) In vivo drug plasma concentration‐time profiles in C57BL/6 mice received i.v. injection of NPs/CAL and free CAL (at a CAL equivalent dose of 5 mg/kg). i.v., intravenous.

To evaluate the targeting ability of the nanomedicine, we tracked its biodistribution through fluorescence imaging. The tissue distribution was validated using NPs attached with the hydrophobic fluorochrome 1,1‐dioctadecyl‐3,3,3,3‐tetramethylindocarbocyanine iodide (DiI) instead of NPs/CAL. As depicted in Figure 3A and B, we found significantly higher accumulation of NPs/DiI in the liver than that in other organs and the fluorescence emission measured by *ex vivo* imaging reached a peak in livers at 8 h, which may be due to the natural advantage of passive accumulation of nanomedicine in the liver, which was conducive to increasing the concentration of CAL in the liver.

We then further evaluated whether the polymeric micelles were located in HSCs. We injected NPs/DiI into healthy mice through the tail vein and then collected the liver tissues of the mice 24 h later. After the liver samples were embedded and sectioned, immunofluorescence staining was performed with an anti‐desmin antibody, a biomarker of HSC. As shown in Figure S7, strong yellow signals were found to appear in HSCs, which was the result of the fluorescent color merging of DiI (red) and desmin (green). Taken together, these results clearly indicated that NPs/CAL had a high liver accumulation and could be taken up by HSCs in vivo.

To verify whether our NPs/CAL‐formulated NP scaffold enabled CAL circulation to be extended in the blood, we performed pharmacokinetic analysis by a single intravenous (i.v.) injection of NPs/CAL or free CAL in C57BL/6 mice. The pharmacokinetic characteristics of NPs/CAL and free CAL are shown in Figure 3C. The elimination phase of free Cal appeared steeper than that of Cal in NPs/CAL. As expected, in comparison with the clearance rate of free CAL, NPs/CAL greatly extended the blood circulation time of the free CAL agent. For instance, at 2 h, the ratio of plasma CAL concentration in the mice receiving NPs/CAL to plasma CAL concentration in the mice receiving free CAL was approximately 4.62 times, and at 6 h, the ratio rose to 6.47 times. In addition, the areas under the concentration–time curve (AUC_0‐t_) for NPs/CAL was 13 times higher than that of the free CAL‐dosed group. In summary, the above results indicated that NPs improved the persistence of CAL in the systemic circulation.

### 2.4 NPs/CAL attenuated carbon tetrachloride (CCl_4_)‐induced liver fibrosis

**FIGURE 4:**
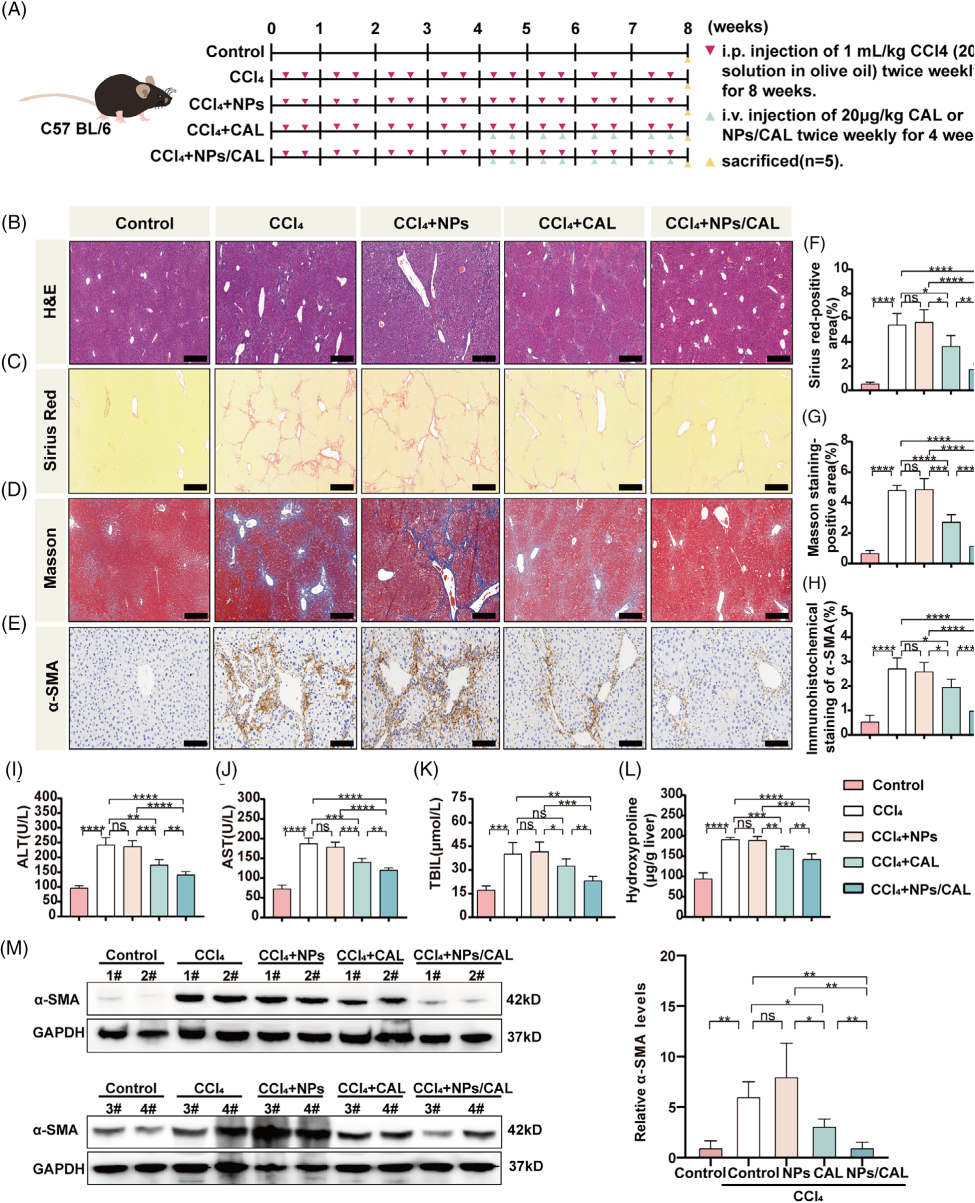
NPs/CAL dampened liver injury and fibrosis in a CCl_4_‐induced mouse model. (A) Schematic flow chart of the in vivo experiment. Mice (*n* = 5/group) received either vehicle or 1 mL/kg CCl_4_ (20% solution in olive oil) intraperitoneally twice weekly for 8 weeks. CAL or NPs/CAL (20 μg/kg body weight) was injected intravenously twice weekly for 4 weeks, commencing 4 weeks after the first dose of CCl_4_. (B–E) Liver tissues were stained using H&E, Sirius red, Masson (scale bar = 200 μm) and immunohistochemical staining for α‐SMA (scale bar = 100 μm). (F–H) Quantification of positive staining areas. (I–K) Serum levels of ALT, AST, and TBIL. (L) Hepatic hydroxyproline content. (M) Immunoblotting confirmation of α‐SMA in harvested liver tissues of the mice. The results shown are mean ± SD. *p < 0.05; **p < 0.01; ***p < 0.001; ****p < 0.0001; ns, no significant difference.

To investigate whether NPs/CAL can ameliorate fibrotic foci in vivo, we generated a mouse liver fibrosis model by intraperitoneal (i.p.) injection of 20% CCl_4_ for 8 weeks. C57BL/6 mice were treated as described in Figure 4A. As evidenced by hematoxylin–eosin (H&E), Sirius red, and Masson staining (Figure 4B, C, D, F, G), higher expression of collagen deposition in the liver was observed in mice that received either CCl_4_ or CCl_4_+NPs compared with controls. However, there was no difference in the accumulation of collagen between CCl_4_‐injured mice and CCl_4_+NPs mice. Interestingly, injection of NPs/CAL into the tail vein of CCl_4_‐injured mice substantially alleviated hepatic lesions and collagen accumulation in the liver tissues, with significantly stronger potency than CAL. Additionally, we examined the hydroxyproline content of liver tissue, which is a major component of collagen and is an index for collagen metabolism and the degree of fibrosis.^18^, ^19^ As shown in Figure 4L, the content of hydroxyproline was markedly decreased when mice were treated with NPs/CAL compared with the CAL group (*p*  <  0.01). In terms of the results of immunochemical staining (Figure 4E, H), the α‐SMA was dramatically decreased in the NPs/CAL group compared to model groups and the CAL group. In concordance, we also observed that the protein signal of α‐SMA was weaker in the NPs/CAL group than that in CCl_4_‐injured mice with CAL treatment according to the immunoblotting result (Figure4M). As depicted in Figure 4I, J, and K, we then observed that the contents of serum alanine aminotransferase (ALT), aspartate aminotransferase (AST), and total bilirubin (TBIL) were all elevated remarkably in either the CCl_4_ or CCl_4_+NPs group when the control group was included for comparison purpose. NPs/CAL treatment prominently reduced the levels of these biomarkers, moreover, the nanodrug displayed better results than CAL (*p* <  0.01). Taken together, all these data suggested that NPs/CAL not only effectively alleviated liver fibrosis but also protected the liver from impairment.

### 2.5 NPs/CAL exhibited a good biosafety

**FIGURE 5:**
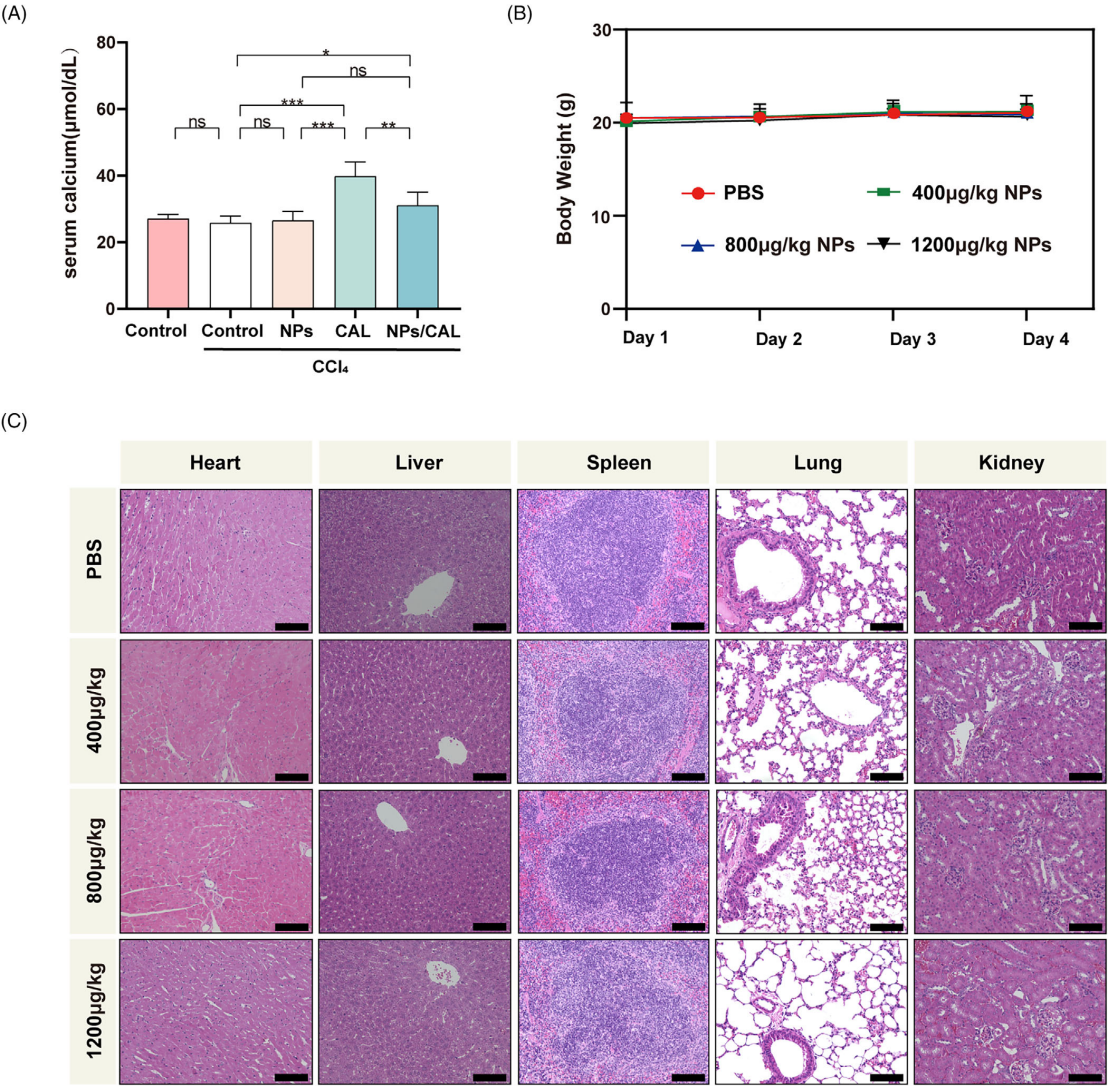
Evaluation of the safety profiles of nanomedicines in animals. (A) The serum calcium concentration of NPs/CAL compared to free CAL in CCl_4_‐induced liver fibrosis was determined using a micro blood calcium concentration assay kit. *n* = 5/group. (B) Daily body weight of mice (*n* = 3/group) receiving i.v. injection of NPs at three different concentrations once a day for 3 consecutive days. (C) Representative of H&E staining of major organ sections from mice (*n* = 3/group), scale bar = 100 μm. The results shown are mean ± SD. *p < 0.05; **p < 0.01; ***p < 0.001; ****p < 0.0001; ns, no significant difference. i.v., intravenous.

CAL may cause the risk of hypercalcemia that imposes restrictions on the application of its maximal dose in clinical practice. However, we hypothesized that our strategy was able to alleviate the serum calcium caused by the side effect of free CAL. In order to verify this assumption, we collected plasma from CCl_4_‐injured mice receiving NPs/CAL or free CAL at the end of an 8‐week model of liver fibrosis and then detected the serum calcium concentration. As the data illustrated in Figure 5A, CCl_4_‐injured mice receiving free CAL exhibited significantly elevated serum calcium levels (39.88 ± 4.27 μmol/dL) compared with those of the control and model groups. This result was in agreement with the clinical observation that CAL could cause the elevation of serum calcium levels. However, to our delight, the serum calcium concentration of CCl_4_‐injured mice following NPs/CAL treatment was proven to be manifestly lower than that of the CAL‐dosed group (*p*  <  0.01). This discrepancy avoided the potential risk of organ damage and attested to the biosafety of NPs/CAL in vivo.

The biosafety study of NPs was further conducted by intravenously injecting the NPs at concentrations of 0, 400, 800, and 1200 μg/kg into the tail vein of healthy mice once a day for 3 consecutive days. During this period, body weight changes were measured daily in each group. We found that different concentrations of NPs did not cause obvious weight loss in each group of mice (Figure 5B). Next, we collected the major organs in healthy mice and conducted a histological study. The representative images depicting H&E staining of the major organs are presented in Figure 5C. Obviously, the results revealed that no injury was observed in the major organs in normal mice treated with different concentrations of polymeric micelles, and the histological characteristics were no different from those in PBS‐treated mice. Therefore, all these observations indicated that NPs/CAL was a safe nanotherapy and that the hypercalcemia of free CAL obtained using our approach was ameliorated.

## 3 DISCUSSION

CAL has been extensively studied as a synthetic VDR agonist that can neutralize TGF‐β1 and reduce liver fibrosis. The differential expression of VDR in liver parenchymal and nonparenchymal cells also determined that CAL could restrict this inhibition only to VDR‐positive cells of the liver (mainly in nonparenchymal cells), rather than unnecessarily blocking TGF‐β1 in nondiseased sites, suggesting a safer antifibrotic strategy.^13^, ^20^ Despite this potency, CAL‐based therapy has achieved only limited improvements in the clinic. Currently, CAL is only approved by the U.S. Food and Drug Administration as a specific drug for psoriasis.^21^ CAL is extremely insoluble in aqueous solutions, and its solubility in solvent can only be increased by the action of some surfactants. Notably, in addition to the liver, VDR is also widely distributed in other tissues, which reduces the amount of CAL reaching the target site and results in possible adverse effects, such as hypercalcemia.

Nanomedicine may provide a feasible solution to the above‐mentioned problems that limit the clinical application of CAL. In this context, our group synthesized a self‐assembled polymeric nanocarrier for delivering CAL to fibrotic lesions, which retained pharmacological efficacy while improving safety. Molecular simulations found high binding energy of cholesterol to CAL, and then, we incorporated this planar hydrophobic stabilizer into the polymer framework to ensure efficient encapsulation of CAL. In a CCl_4_‐induced liver fibrosis mouse model, we found that NPs/CAL therapy not only effectively prevented the progression of liver fibrosis but also showed significant potency in reducing liver injury. However, the efficacy of NPs/CAL in other mouse models of liver fibrosis remains to be determined, such as bile duct ligation, methionine‐ and choline‐deficient diet, and choline‐deficient amino acid‐defined diet models.

PEGylation technology is often used to create a “stealth” outer shell for nanoparticles, which significantly improves the solubility of drug molecules, reduces immunogenicity, prolongs half‐life, and so on.^22^ Our study found that the long‐term circulation of CAL was improved with the help of PEGylated nanoparticles, and that NPs showed the best accumulation in the liver of mice and were able to quickly and efficiently release cargo in HSCs. Our future work will focus on coupling targeting ligands on the surface of the nanoparticles to further improve their cell or tissue targeting specificity.

To date, many inorganic or organic nanoparticles have been described as promising and useful strategies for the diagnosis and treatment of hepatic fibrogenesis.^23^, ^24^, ^25^, ^26^ For example, both titanium dioxide nanoparticles and silica nanoparticles were able to exhibit potential antifibrotic activity in vitro.^27^ However, the toxicity of nanomaterials should be considered before the widespread use of nano‐based delivery systems, especially inorganic nanoparticles.^28^, ^29^ In this regard, the risk associated with NPs/CAL seemed to be less problematic because they were made from a highly biocompatible polymer PEG. Further exploration revealed that NPs/CAL in vivo did not cause an obvious increase in serum calcium levels. The high degree of separation of the antifibrosis effect of NPs/CAL from its calcemic effect might be due to the fact that CAL was primarily transported to the liver by nanocarriers and ultimately utilized by liver cells, so as to prevent a large amount of CAL from reaching other organs, such as the small intestine and bone, to exert its biological effect. Thus, the intestinal absorption of calcium and phosphorus and the release of bone calcium were effectively reduced.^30^ Overall, the preclinical data of the injectable nanomedicine fully displayed a design strategy for simultaneously improving the efficacy and safety of CAL against liver fibrosis.

## 4 MATERIALS AND METHODS

### 4.1 Chemicals and instruments

CAL was acquired from Puyi Chemical Co. Azobisisobutyronitrile (AIBN > 98%), 2‐hydroxyethyl methacrylate and cholesteryl chloroformate were obtained from Aladdin. All other chemical reagents and solvents were purchased from Macklin Chemical Reagent Co., Ltd. Elemental mapping images were photographed by TEM using an HT‐7700 electron microscope (Hitachi Ltd.) and an X‐MAXn65T CCD camera (Oxford Instruments). The size distribution and zeta potential of nanoparticles were measured using a Malvern Zetasizer Nano‐ZS for DLS. The Malvern Zetasizer Nano‐ZS system consisted of a laser operating at 633 nm and a multi‐tau digital correlator electronics system.

### 4.2 Synthesis of PEG_5K_‐P(HEMACHL)_7K_ and NPs/CAL

PEG_5K_‐P(HEMACHL)_7K_ was synthesized by RAFT. Briefly, 213 mg PEG‐PETTC, 300 mg HEMACHL, and 3.3 mg AIBN were placed into a Schlenk flask, which connected a set of degassing devices. After 1 mL of dried DMF and 1 mL of dried 1,4‐dioxane were added, the flask underwent a freeze‐evacuate‐thaw process of degassing three times. The solution was kept at 70°C for 16 h. Then, 10 mL of DMF was added, and the solution obtained was dialyzed against 3 × 0.25 L of DMF, and 1 L of ddH_2_O for three times. Finally, PEG_5K_‐P(HEMACHL)_7K_ was obtained through lyophilization (white powder, yield of 54%).

The NPs/CAL was manufactured with a similar method. Briefly, 5 mg PEG_5K_‐P(HEMACHL)_7K_ and an amount of CAL were dissolved in 1.5 mL of DMF, and 1.5 mL of water was added dropwise and then stirred completely through a magnetic stirrer at an appropriate rate. After homogenization by sonication, we loaded a dialysis bag (Mw = 3500 Da) with the solution against 3 × 1 L of ddH_2_O for 12 h of dialysis. The obtained solution was concentrated by rotary evaporation, homogenized by sonication, and filtered to screen the bacteria out and stored in 4°C before use. NPs/Cor6 and NPs/DiI were fabricated similarly.

### 4.3 Detection of DL, encapsulation rate, and CMC

The DL and encapsulation rate were performed by HPLC. After the baseline was basically balanced, an automatic injection of a 20 μL sample at 30°C was conducted. The mobile phase was a mixture of pure methanol: 0.1% trifluoroacetic acid aqueous solution = 85:15 (v/v %). The UV detector was used to detect CAL at 265 nm wavelength. The CMC of nanoparticles was detected as previously described.^31^

### 4.4 in vitro CAL release kinetics

NPs/CAL was incubated in the appropriate buffer solution (100 mM Tris buffer, 100 mM NaCl, pH = 7.4, 1% Tween 80) at 37°C and added to a dialysis bag (molecular weight = 3500 Da). Then, the sealed dialysis bag was incubated in 50 mL of buffer solution with agitation for 24 h. At 0, 0.5, 2, 6, 12, and 24 h, 0.1 mL solution was extracted and replaced with an equal volume of fresh medium. The concentrations of CAL in samples were determined by the HPLC method described above.

### 4.5 Hemolytic activity assay

Hemolytic activity was assessed as previously reported.^32^, ^33^ In brief, 200 μL of diluted blood was added to 800 μL PBS (pH 7.4) which contained different amounts of NPs (25, 50, 75, and 100 μg). After an additional 4 h incubation at 37°C, the samples were centrifuged and 200 μL of each supernatant was added to a single well of the 96‐well plate. We selected the optical density (OD) near the 540 nm wavelength by a microplate reader to quantitatively analyze the hemolytic activity. Relative hemolysis rate (%) was calculated as [(As‐An)/(Ap‐An)] × 100%, where As represented the OD of the sample, An represented the negative control (the OD of blood mixed PBS), and Ap represented the positive control (the OD of PBS containing Triton X‐100).

### 4.6 Cell‐based evaluative experiments

#### 4.6.1 Cell culture and drug treatment

L‐02, LX‐2, and HSC‐T6 cells were cultured in Dulbecco's modified Eagle's medium (DMEM; HyCloneTM #AE29431636) with 10% fetal bovine serum (FBS; Corning #35081006) at 37°C in a 5% CO_2_ incubator. LX‐2 cells were seeded in 6‐well plates and incubated with 100 nM CAL or NPs/CAL and 5 ng/mL TGF‐β1 (R&D Systems #240‐B‐002) for 48 h.

#### 4.6.2 Cell survival assay

The chemical MTT was used for cell survival assay as previously described.^16^

#### 4.6.3 Cellular uptake and intracellular distribution

LX‐2 cells were incubated with NPs/Cor6 for 0.5, 2, and 4 h, respectively. The cell entry rate of nanoparticles was determined using flow cytometry. Intracellular distribution was observed by a Leica SP8 DIVE CLSM (Leica, Germany). Lysosomes were labeled with LysoTracker Red DND‐99 (100 nM, Thermo Fisher Scientific #L7528), and the nucleus was labeled with Hoechst 33342 (Thermo Fisher Scientific #R37605).

### 4.7 Animal‐based studies

#### 4.7.1 Murine liver fibrosis model construction and treatment

C57BL/6 male mice (8 weeks old, 20−25 g) were obtained from Shanghai SLAC Laboratory Animal Co., Ltd. All works and animal protocols performed on animals were approved by the Ethics Review and Scientific Investigation Board of the First Affiliated Hospital, Zhejiang University (No. 20201086). To induce liver fibrosis, mice were administered with 1 mL/kg CCl_4_ (Adamas‐beta #65805; 20% solution in olive oil from Sangon Biotech Co., Ltd., # A502795) by i.p. injection twice weekly for 8 weeks. The control mice received a vehicle instead. CAL or NPs/CAL (CAL concentration, 20 μg/kg body weight) was injected intravenously twice a week for a total of eight doses, commencing 4 weeks after the first i.p. injection of CCl_4_. Mice were sacrificed 3 days after the animals had received their last injection of CCl_4_, and then, further analysis was conducted on liver tissue and blood samples.

#### 4.7.2 Tissue distribution study

We intravenously administered NPs/DiI into C57BL/6 normal mice. At 2, 8, 24, 32, and 48 h post‐administration, mice (*n* = 3 at each time point) were terminated, and the liver, kidney, spleen, lung, and heart were collected for the determination of the fluorescence emission by *ex vivo* imaging.

#### 4.7.3 Pharmacokinetic study

After a single i.v. injection of NPs/CAL or free CAL (at a CAL equivalent dose of 5 mg/kg) into the tail vein of C57BL/6 mice (*n* = 3/group), orbital blood sampling was performed at the indicated time points. Subsequently, 50 μL of plasma sample at each time point was then transferred to a 1.5 mL centrifuge tube containing 150 μL of methanol and vortically mixed for 5 min, followed by high‐speed centrifugation. Eventually, the plasma drug concentration was analyzed by HPLC‐mass spectrometry.

#### 4.7.4 Immunohistochemistry analysis

Immunohistochemistry was used to detect in vivo α‐SMA expression which was performed according to a standard protocol.^34^ Ten random fields were selected for each section and quantified by Image‐Pro Plus software.

#### 4.7.5 Lab data detection of blood plasma and serum samples

ALT, AST, and TBIL were detected using commercial kits (Fujifilm #3250, #3150, #2150). The serum calcium concentration was detected by a micro blood calcium concentration assay kit (Beijing Solarbio Science & Technology Co., Ltd., #BC0725).

#### 4.7.6 Hydroxyproline content measurement

According to the experimental steps described in the instructions of a kit (Beijing Solarbio Science & Technology Co., Ltd., #BC0255), we evaluated the content of hydroxyproline in liver tissue.

#### 4.7.7 Western blotting

Western blotting was performed as previously described.^35^ The specific primary antibodies used here included α‐SMA (1:1000, CST #19245), and GAPDH (1:1000, CST #5174).

#### 4.7.8 Immunofluorescence staining

Mice were injected with a single dose of NPs/DiI through the tail vein. At 24 h post‐administration, liver tissue samples were collected and paraffin‐embedded. Sections were incubated at 4°C overnight with a rabbit anti‐desmin antibody (1:200, proteintech #16520‐1‐AP) and then with an AlexaFluor 488‐labeled goat anti‐rabbit secondary antibody at room temperature for about 1 h. Next, nuclei were labeled by staining with 4,6‐diamidino‐2‐phenylindole (DAPI). Finally, the frozen sections were photographed and scanned for colocalization analysis.

### 4.8 Statistical analysis

All statistical analyses were calculated using the SPSS 22.0 program. Values were expressed as mean ± SD. Differences between multiple groups of data were analyzed by one‐way analysis of variance (ANOVA) with Bonferroni correction. Generally, *p* < 0.05 was considered statistically significant.

## AUTHOR CONTRIBUTIONS

Yina Zhang: Conceptualization, data curation, formal analysis, visualization, investigation, project administration, writing—original draft. Liying Wang: Investigation, conceptualization, methodology, data curation, supervision, writing—original draft. Jiajia Shao: Data curation, formal analysis, investigation, writing—review and editing. Yanning Liu: Writing—review and editing, investigation. Yining Lu: Formal analysis, investigation. Jing Yang, Siduo Xu, Jingkang Zhang, and Minwei Li: Investigation. Xiangrui Liu: Conceptualization, investigation, project administration, funding acquisition, supervision, writing—review and editing. Min Zheng: Conceptualization, funding acquisition, supervision, writing—review and editing. All authors have read and approved the final manuscript.

## CONFLICT OF INTEREST STATEMENT

The authors have no conflict of interest to declare.

## ETHICS APPROVAL AND CONSENT TO PARTICIPATE

This research was allowed by the Medical Ethics Committee of the First Affiliated Hospital of Zhejiang University. All protocols of animal experiments were approved by the Animal Experimental Ethics Committee of the First Affiliated Hospital of Zhejiang University (No. 20201086).

## Supporting information

Supporting Information

## Data Availability

The datasets analyzed during the current study are available from the corresponding author on reasonable request.

## References

[1] Loureiro D , Tout I , Narguet S , Benazzouz SM , Mansouri A , Asselah T . miRNAs as potential biomarkers for viral hepatitis B and C. Viruses. 2020;12(12):1440. 33327640 10.3390/v12121440 PMC7765125

[2] Rockey DC , Bell PD , Hill JA . Fibrosis–a common pathway to organ injury and failure. N Engl J Med. 2015;372(12):1138‐1149. 25785971 10.1056/NEJMra1300575

[3] Tsochatzis EA , Bosch J , Burroughs AK . Liver cirrhosis. Lancet. 2014;383(9930):1749‐1761. 24480518 10.1016/S0140-6736(14)60121-5

[4] Asrani SK , Devarbhavi H , Eaton J , Kamath PS . Burden of liver diseases in the world. J Hepatol. 2019;70(1):151‐171. 30266282 10.1016/j.jhep.2018.09.014

[5] Tateishi R , Uchino K , Fujiwara N , et al. A nationwide survey on non‐B, non‐C hepatocellular carcinoma in Japan: 2011–2015 update. J Gastroenterol. 2019;54(4):367‐376. 30498904 10.1007/s00535-018-1532-5 PMC6437291

[6] GBDRF Collaborators . Global, regional, and national comparative risk assessment of 79 behavioural, environmental and occupational, and metabolic risks or clusters of risks, 1990–2015: a systematic analysis for the Global Burden of Disease Study 2015. Lancet. 2016;388(10053):1659‐1724. 27733284 10.1016/S0140-6736(16)31679-8 PMC5388856

[7] Kisseleva T . The origin of fibrogenic myofibroblasts in fibrotic liver. Hepatology. 2017;65(3):1039‐1043. 27859502 10.1002/hep.28948 PMC5476301

[8] Dawood RM , El‐Meguid MA , Salum GM , El Awady MK . Key players of hepatic fibrosis. J Interferon Cytokine Res. 2020;40(10):472‐489. 32845785 10.1089/jir.2020.0059

[9] Seki E , Brenner DA . Recent advancement of molecular mechanisms of liver fibrosis. J Hepatobiliary Pancreat Sci. 2015;22(7):512‐518. 25869468 10.1002/jhbp.245 PMC4668270

[10] Watanabe Y , Tsuchiya A , Terai S . The development of mesenchymal stem cell therapy in the present, and the perspective of cell‐free therapy in the future. Clin Mol Hepatol. 2021;27(1):70‐80. 33317249 10.3350/cmh.2020.0194 PMC7820202

[11] Zhao Z , Lin CY , Cheng K . siRNA‐ and miRNA‐based therapeutics for liver fibrosis. Transl Res. 2019;214:17‐29. 31476281 10.1016/j.trsl.2019.07.007 PMC6848786

[12] Kissmeyer AM , Binderup L . Calcipotriol (MC 903): pharmacokinetics in rats and biological activities of metabolites. A comparative study with 1,25(OH)2D3. Biochem Pharmacol. 1991;41(11):1601‐1606. 2043150 10.1016/0006-2952(91)90160-7

[13] Ding N , Yu RT , Subramaniam N , et al. A vitamin D receptor/SMAD genomic circuit gates hepatic fibrotic response. Cell. 2013;153(3):601‐613. 23622244 10.1016/j.cell.2013.03.028 PMC3673534

[14] Wahsh E , Abu‐Elsaad N , El‐Karef A , Ibrahim T . The vitamin D receptor agonist, calcipotriol, modulates fibrogenic pathways mitigating liver fibrosis in‐vivo: an experimental study. Eur J Pharmacol. 2016;789:362‐369. 27477355 10.1016/j.ejphar.2016.07.052

[15] Wang X , Wang G , Qu J , Yuan Z , Pan R , Li K . Calcipotriol inhibits NLRP3 signal through YAP1 activation to alleviate cholestatic liver injury and fibrosis. Front Pharmacol. 2020;11:200. 32296329 10.3389/fphar.2020.00200 PMC7136474

[16] Wang L , Liu Z , Zhou Q , et al. Prodrug nanoparticles rationally integrating stroma modification and chemotherapy to treat metastatic pancreatic cancer. Biomaterials. 2021;278:121176. 34656882 10.1016/j.biomaterials.2021.121176

[17] Jones ER , Semsarilar M , Blanazs A , Armes SP . Efficient synthesis of amine‐functional diblock copolymer nanoparticles via RAFT dispersion polymerization of benzyl methacrylate in alcoholic media. Macromolecules. 2012;45(12):5091‐5098.

[18] Udenfriend S . Formation of hydroxyproline in collagen. Science. 1966;152(3727):1335‐1340. 5327887 10.1126/science.152.3727.1335

[19] Toyoki Y , Sasaki M , Narumi S , Yoshihara S , Morita T , Konn M . Semiquantitative evaluation of hepatic fibrosis by measuring tissue hydroxyproline. Hepatogastroenterology. 1998;45(24):2261‐2264. 9951907

[20] Gascon‐Barre M , Demers C , Mirshahi A , Neron S , Zalzal S , Nanci A . The normal liver harbors the vitamin D nuclear receptor in nonparenchymal and biliary epithelial cells. Hepatology. 2003;37(5):1034‐1042. 12717384 10.1053/jhep.2003.50176

[21] Guenther LC . Treatments for scalp psoriasis with emphasis on calcipotriol plus betamethasone dipropionate gel (Xamiol). Skin Therapy Lett. 2009;14(4):1‐4. 19585059

[22] Suk JS , Xu Q , Kim N , Hanes J , Ensign LM . PEGylation as a strategy for improving nanoparticle‐based drug and gene delivery. Adv Drug Deliv Rev. 2016;99:28‐51. 26456916 10.1016/j.addr.2015.09.012 PMC4798869

[23] Li Y , Shang W , Liang X , et al. The diagnosis of hepatic fibrosis by magnetic resonance and near‐infrared imaging using dual‐modality nanoparticles. RSC Adv. 2018;8(12):6699‐6708. 35540380 10.1039/c7ra10847h PMC9078292

[24] Duong HT , Dong Z , Su L , et al. The use of nanoparticles to deliver nitric oxide to hepatic stellate cells for treating liver fibrosis and portal hypertension. Small. 2015;11(19):2291‐2304. 25641921 10.1002/smll.201402870

[25] Jimenez Calvente C , Sehgal A , Popov Y , et al. Specific hepatic delivery of procollagen alpha1(I) small interfering RNA in lipid‐like nanoparticles resolves liver fibrosis. Hepatology. 2015;62(4):1285‐1297. 26096209 10.1002/hep.27936 PMC4589454

[26] Li LJ , Wang HY , Ong ZY , et al. Polymer‐ and lipid‐based nanoparticle therapeutics for the treatment of liver diseases. Nano Today. 2010;5(4):296‐312.

[27] Peng F , Tee JK , Setyawati MI , et al. Inorganic nanomaterials as highly efficient inhibitors of cellular hepatic fibrosis. ACS Appl Mater Interfaces. 2018;10(38):31938‐31946. 30156820 10.1021/acsami.8b10527

[28] Soenen SJ , Rivera‐Gil P , Montenegro JM , Parak WJ , De Smedt SC , Braeckmans K . Cellular toxicity of inorganic nanoparticles: common aspects and guidelines for improved nanotoxicity evaluation. Nano Today. 2011;6(5):446‐465.

[29] Soenen SJ , Manshian B , Montenegro JM , et al. Cytotoxic effects of gold nanoparticles: a multiparametric study. ACS Nano. 2012;6(7):5767‐5783. 22659047 10.1021/nn301714n

[30] Tebben PJ , Singh RJ , Kumar R . Vitamin D‐mediated hypercalcemia: mechanisms, diagnosis, and treatment. Endocr Rev. 2016;37(5):521‐547. 27588937 10.1210/er.2016-1070 PMC5045493

[31] Wang L , Liu X , Zhou Q , et al. Terminating the criminal collaboration in pancreatic cancer: nanoparticle‐based synergistic therapy for overcoming fibroblast‐induced drug resistance. Biomaterials. 2017;144:105‐118. 28837958 10.1016/j.biomaterials.2017.08.002

[32] Xie RL , Jang YJ , Xing L , et al. A novel potential biocompatible hyperbranched polyspermine for efficient lung cancer gene therapy. Int J Pharm. 2015;478(1):19‐30. 25448566 10.1016/j.ijpharm.2014.11.014

[33] Fan QQ , Zhang CL , Qiao JB , et al. Extracellular matrix‐penetrating nanodrill micelles for liver fibrosis therapy. Biomaterials. 2020;230:119616. 31837823 10.1016/j.biomaterials.2019.119616

[34] Lv Q , Wang W , Xue J , et al. DEDD interacts with PI3KC3 to activate autophagy and attenuate epithelial‐mesenchymal transition in human breast cancer. Cancer Res. 2012;72(13):3238‐3250. 22719072 10.1158/0008-5472.CAN-11-3832

[35] Wang Y , Wen H , Fu J , et al. Hepatocyte TNF receptor‐associated factor 6 aggravates hepatic inflammation and fibrosis by promoting lysine 6‐linked polyubiquitination of apoptosis signal‐regulating kinase 1. Hepatology. 2020;71(1):93‐111. 31222801 10.1002/hep.30822

