# Supplementary material for: Nano‐calcipotriol as a potent anti‐hepatic fibrosis agent
Source: MedComm (2020). 2023 Aug 26;4(5):e354. doi: 10.1002/mco2.354 (PMC10458662; doi:10.1002/mco2.354)

**Supporting Information**

**Nano-calcipotriol as a potent anti-hepatic fibrosis agent**

Yina Zhang^1#^, Liying Wang^2,3,4#^, Jiajia Shao^1#^, Yanning Liu^1^, Yining Lu^1^, Jing Yang^1^, Siduo Xu^1^, Jingkang Zhang^1^, Minwei Li^1^, Xiangrui Liu^2,4,5*^, and Min Zheng^1*^

^1^ State Key Laboratory for Diagnosis and Treatment of Infectious Diseases, National Clinical Research Center for Infectious Diseases, Collaborative Innovation Center for Diagnosis and Treatment of Infectious Diseases, The First Affiliated Hospital, College of Medicine, Zhejiang University, 79 Qingchun Road, Hangzhou 310003, China

^2^ Department of Pharmacology and Department of Gastroenterology of the Second Affiliated Hospital, Zhejiang University School of Medicine, Hangzhou, 310058, China

^3^ Department of General Surgery, Sir Run Run Shaw Hospital, Zhejiang University School of Medicine, Hangzhou, 310058, China

^4^ Key Laboratory of Biomass Chemical Engineering of Ministry of Education and Center for Bionanoengineering, College of Chemical and Biological Engineering, Zhejiang University, Hangzhou, 310027, China

^5^ Cancer Center, Zhejiang University, Hangzhou, Zhejiang, 310058, China

^#^ Yina Zhang, Liying Wang and Jiajia Shao contributed equally to this work.

***** Corresponding authors:

**Min Zheng**, Ph.D. Professor

E-mail: [minzheng@zju.edu.cn](mailto:minzheng@zju.edu.cn)

**Xiangrui Liu**, Ph.D. Associate Professor

E-mail: [xiangrui@zju.edu.cn](mailto:xiangrui@zju.edu.cn)

**Supplementary Figure Legends**

**Figure S1.** The **^1^**H-NMR spectrum (300 MHz) of cholesterol monomer (CDCl_3_, δ ppm): 0.67-2.42 (m, -CH_3_, -CH(CH_3_)-, -CH-, -CH_2_-), 4.39 (s, 4H, -CO-O-CH_2_-CH_2_-O-CO-), 4.48-4.50 (m, 1H, -CHO-), 5.50-5.70 (m, 1H, -CH=HCH), 6.10-6.20 (m, 1H, -CH=HCH).

**Figure S2.** Gel permeation chromatography of the macroinitiator and PEG_5K_-P(HEMACHL)_7K_ copolymers.

**Figure S3.** Fluorescence emission intensity plot of Nile Red with PEG_5K_-P(HEMACHL)_7K_. The critical micellar concentrations (CMC) of the copolymer was detected by the mutation in the fluorescence emission intensity plot of Nile Red.

**Figure S4.** Zeta potential of NPs and NPs/CAL.

**Figure S5.** Drug release profiles to demonstrate the rate of CAL release from the nanoparticles at 37 °C in a Tris buffer.

**Figure S6.** Hemolytic activity test of different concentrations of NPs on red blood cells (RBCs). RBCs in PBS/Triton X-100 and PBS were designed as positive and negative control, respectively. All error bars correspond to mean ± SD; n=3.

**Figure S7.** Colocalization of DiI-labeled NPs with HSCs in healthy mice. Liver tissues were sectioned and immunostained with an anti-desmin antibody (green) for HSCs and counterstained with the nuclear dye, DAPI (blue). Fluorescent DiI was shown in red. Colocalization was determined as yellow signals corresponding to perfect merges of green and red fluorescence (scale bar = 10 µm).

**Figure S1**


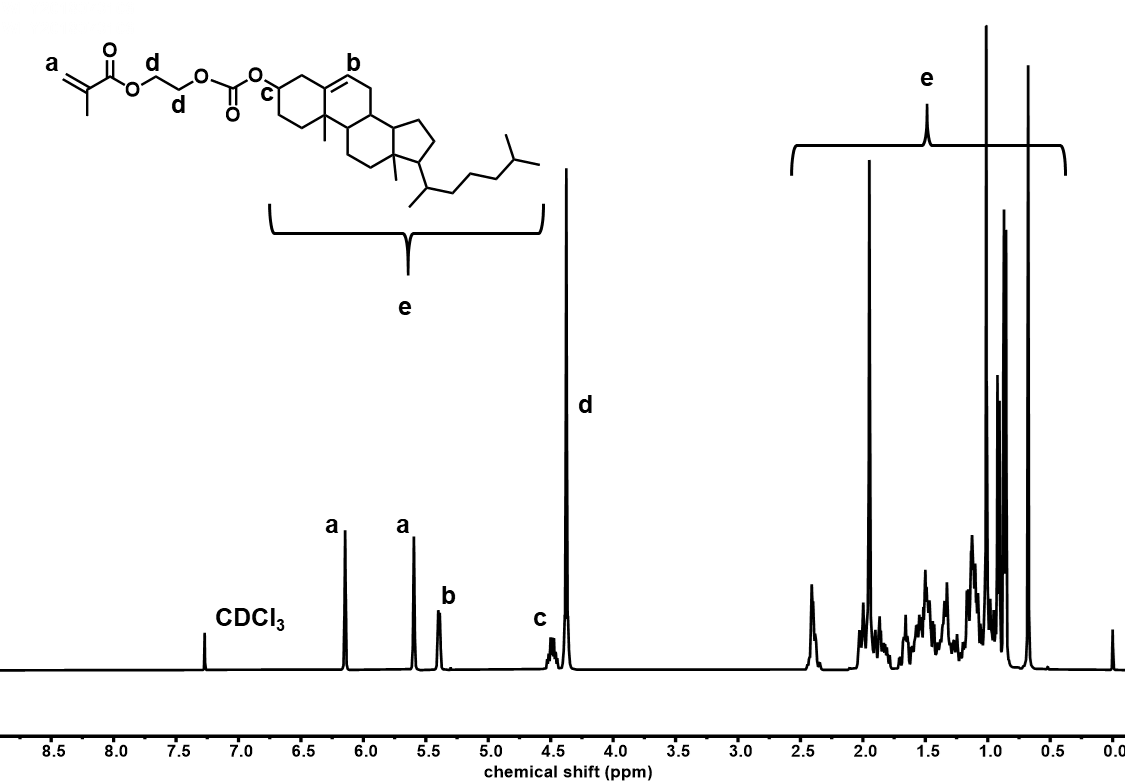


**Figure S2**


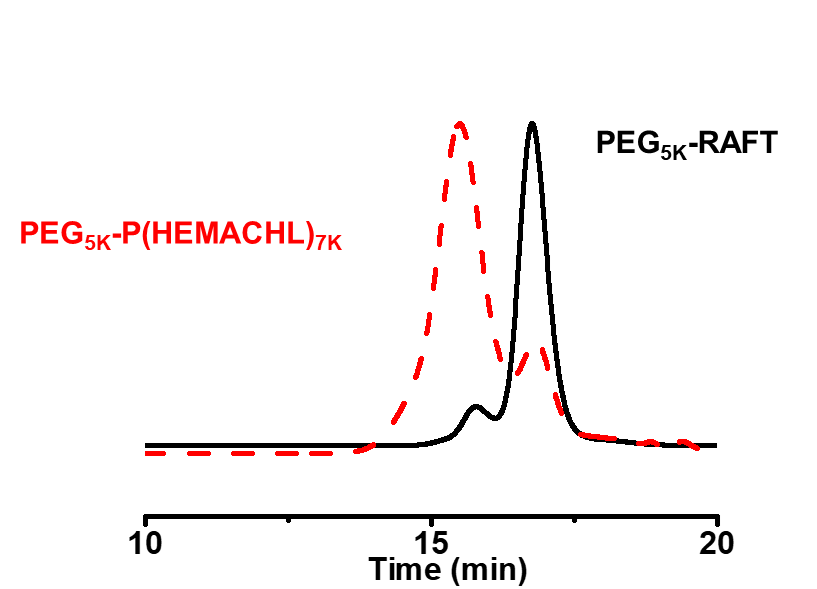


**Figure S3**


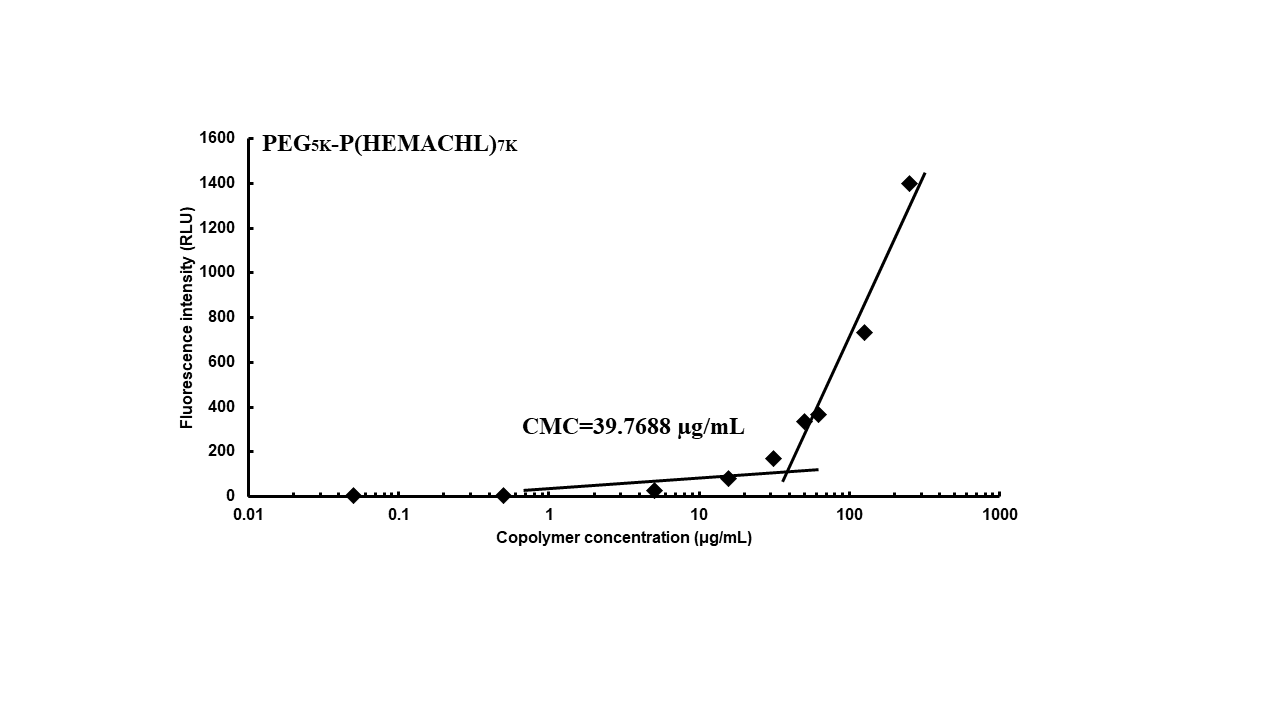


**Figure S4**


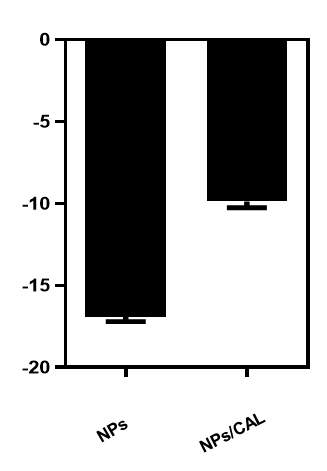


**Figure S5**

**
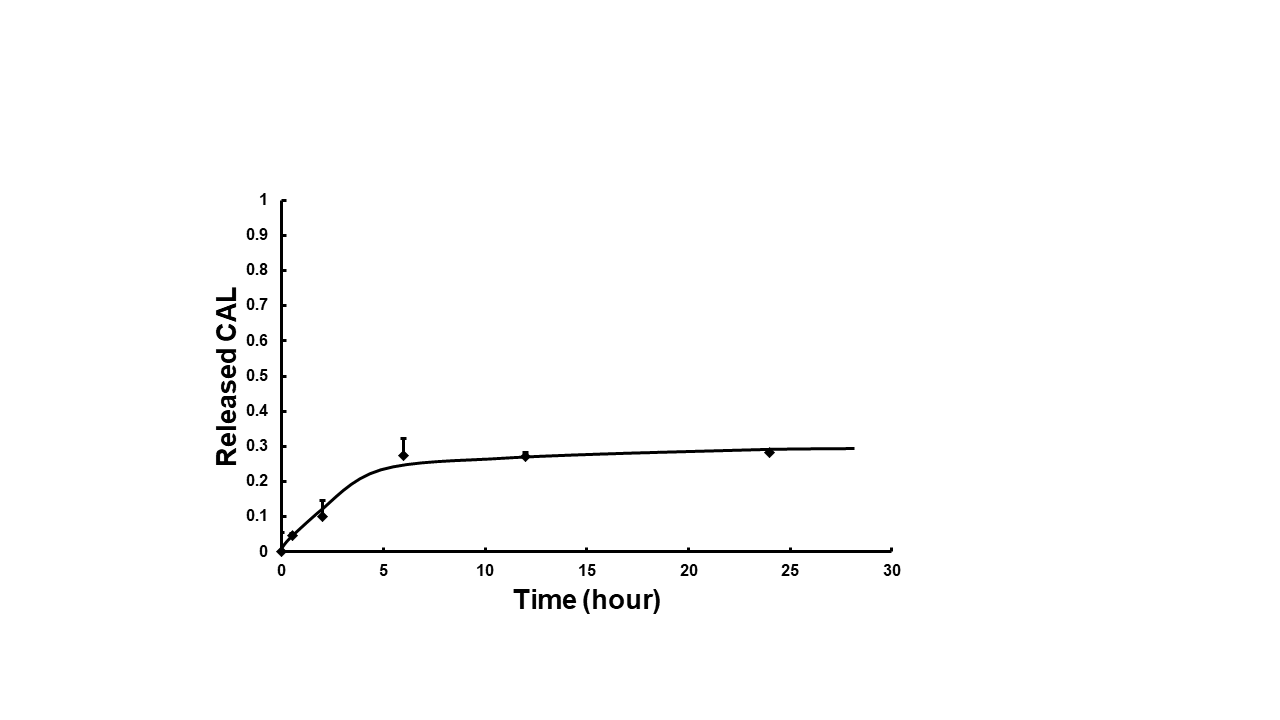
**

**Figure S6**


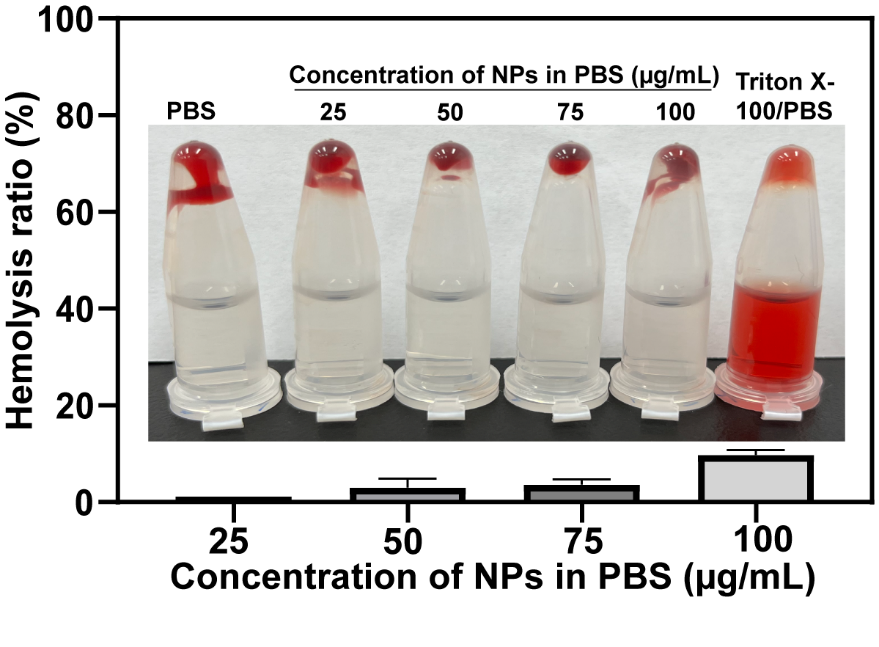


**Figure S7**


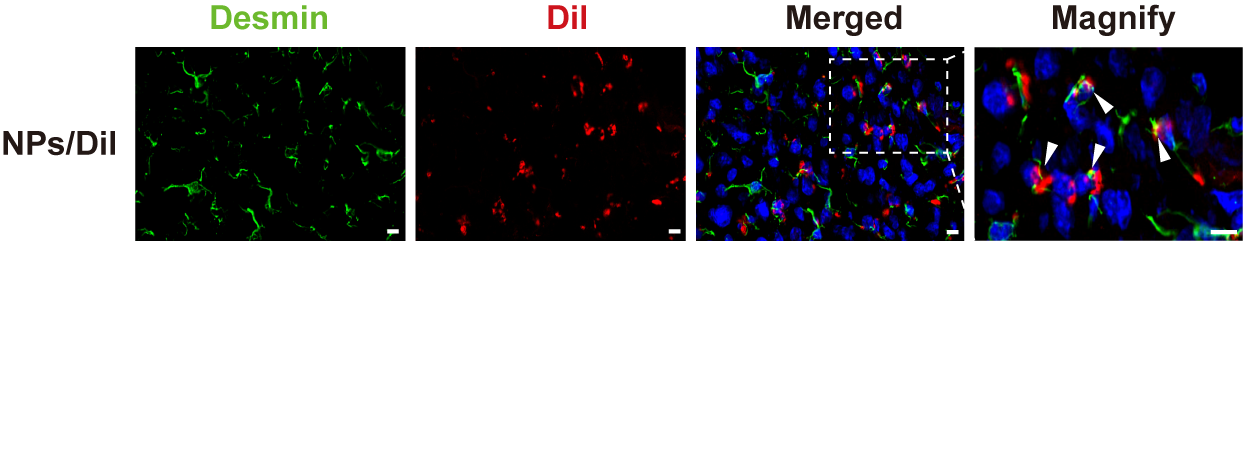

Supplement: Supplementary file 1 — Supporting Information [file MCO2-4-e354-s001.docx]
